# Comparison of Immediate Versus Staged Complete Revascularisation in Patients Presenting With Acute Coronary Syndrome and Multivessel Disease: A Meta-Analysis of Randomized and Non-randomized Studies

**DOI:** 10.7759/cureus.43968

**Published:** 2023-08-23

**Authors:** Anurag Rawat, Sumreen Nazly, Jasvant Kumar, Tayyaba J Khan, Komal Kaur, Gurvir Kaur, Saima Batool, Areeba Khan

**Affiliations:** 1 Interventional Cardiology, Himalayan Institute of Medical Sciences, Baksar Wala, IND; 2 Internal Medicine, University Medical & Dental College Faisalabad, Faisalabad, PAK; 3 Internal Medicine, Chandka Medical College, Larkana, PAK; 4 Internal Medicine, Liaquat University of Medical and Health Sciences, Jamshoro, PAK; 5 Medicine, American University of Antigua, Osburn, ATG; 6 Medicine, Chino Valley Medical Center, Chino, USA; 7 Internal Medicine, Hameed Latif Hospital, Lahore, PAK; 8 Critical Care Medicine, United Medical and Dental College, Karachi, PAK

**Keywords:** systematic review and meta-analysis, multivessel disease, acute coronary syndrome, staged vascularization, immediate vascularization

## Abstract

Acute myocardial infarction is a critical medical condition that poses a significant health burden, leading to substantial morbidity. Despite advancements in medical care, managing this condition is challenging for patients and society. The preferred approach appears to be comprehensive multivessel revascularization, yet the optimal timing remains uncertain. This study aims to compare immediate complete revascularisation and stage complete vascularization in patients presenting with acute coronary syndrome (ACS) and multivessel coronary artery disease (MVD). The Preferred Reporting of Systematic Reviews and Meta-analysis (PRISMA) guidelines conducted the present meta-analysis. A comprehensive literature search was conducted using online databases, including PubMed, and EMBASE from 2010 onwards, to identify articles that compared cardiovascular outcomes between patients undergoing immediate and staged complete revascularization. We also searched Google Scholar for additional studies relevant to the present meta-analysis. The primary outcome assessed in this study was major adverse cardiovascular events (MACE). Secondary outcomes included all-cause mortality, cardiovascular mortality, myocardial infarction (MI), and revascularization. A total of 15 studies fulfilled pre-defined eligibility criteria and were included in the final analysis. Our analysis shows that staged revascularization is associated with improved outcomes in patients with ACS and multivessel CAD, including all-cause mortality and cardiovascular mortality, without increasing the risk of major adverse cardiovascular events, myocardial infarction, and the need for unplanned revascularization.

## Introduction and background

Acute myocardial infarction is a critical medical condition that poses a significant health burden, leading to substantial morbidity. Despite advancements in medical care [1], managing this condition is challenging for patients and society. Percutaneous coronary intervention (PCI) is fundamental in treating Acute Myocardial Infarction (AMI) patients. Notably, over 50% of AMI patients likely have multivessel coronary artery disease (MVD), often linked to unfavorable outcomes [2-3]. Patients with MVD tend to have a less favorable prognosis than those with only one affected vessel [4]. The guidance in ST-elevation myocardial infarction (STEMI) protocols was established based on multiple randomized controlled trials (RCTs) that showed that complete revascularization yields better results than a strategy focusing solely on the culprit lesion, particularly regarding major adverse cardiovascular events (MACE). However, this positive effect was primarily driven by the reduced need for revascularization and the reduction in angina [5-6]. The recent COMPLETE trial, for the first time, demonstrated that complete revascularization has an advantage over the primary endpoint of myocardial infarction (MI) or cardiovascular mortality [7].

The preferred approach appears to be comprehensive multivessel revascularization, yet the optimal timing remains uncertain. This procedure can be conducted either during the index procedure or intervention or in a staged manner. In the latter case, staged revascularization can be performed either during the same hospitalization or even on an outpatient basis. Due to limited data, the STEMI guidelines do not provide specific recommendations regarding the timing of revascularization. According to the SMILE study, the NSTE-ACS guidelines mention that considering complete revascularization during the initial procedure may be contemplated (Class IIB, LOE B) [8-9]. Dangas et al. evaluated the most appropriate timing for staged PCI in patients with MVD, relying on the insights of interventional cardiology specialists. Their findings revealed that around 80% of surveyed interventional cardiologists propose delayed staged PCI for STEMI patients, while 37% advocate a similar approach for patients with non-STEMI (NSTEMI). Regarding the timing of staged PCI, 62% of cardiologists suggested a waiting period of more than two weeks for STEMI patients, and 55% recommended a similar timeframe for NSTEMI patients. The variability in decision-making stems from numerous factors that impact the timing and choice of staged revascularization [10].

This systematic review and meta-analysis aimed to compare immediate complete revascularization during the index procedure versus staged complete revascularisation in patients presenting with ACS (including STEMI and NSTE-ACS) and MVD. Because of the limited data, this systematic review included RCTs and non-randomized trials.

## Review

Methodology

The present meta-analysis was conducted in accordance with the Preferred Reporting of Systematic Review and Meta-analysis (PRISMA) guidelines.

Literature Search

A comprehensive literature search was conducted using online databases, including PubMed, and EMBASE, from 2010 onwards to identify articles that compared cardiovascular outcomes between patients undergoing immediate and staged complete revascularization. We also searched Google Scholar to find additional studies relevant to the objective of this study. Key terms used to search for relevant articles included "immediate revascularization," "staged revascularization," "acute coronary syndrome," and "multivessel disease." We used synonyms, Medical Subject Heading (MeSH) terms to further sensitize the search to identify additional articles; the reference lists of included articles were also manually searched. The literature search was performed independently by two authors.

Study Selection with Inclusion/Exclusion Criteria

Two authors independently screened articles to identify eligible studies. Discrepancies were resolved through consensus and discussion. Studies were considered eligible for inclusion in this meta-analysis if they met the pre-defined criteria: (a) any randomized controlled trial (RCT) or observational study comparing cardiovascular outcomes between immediate and staged complete revascularization in patients with ACS (STEMI or NSTEMI) and MVD, (b) published in the English language, and (c) reported the required outcomes. Studies with a follow-up duration of less than 12 months were excluded. Additionally, articles that included patients with cardiogenic shock were excluded. Case reports, editorials, meta-analyses, and systematic reviews were also excluded.

*Data Extraction, Outcomes, and *Risk of Bias Assessment

Data were extracted from the included studies using a standardized data collection form developed in Microsoft Excel (The Microsoft Corporation). The extracted data included author names, publication years, study designs, sample sizes, ages, genders, diabetes mellitus, hypertension, type of ACS, and dyslipidemia. The primary outcome assessed in this study was major adverse cardiovascular events (MACE). Secondary outcomes included all-cause mortality, cardiovascular mortality, myocardial infarction (MI), and revascularization. Two authors independently performed the risk of bias assessment for each included study. The Cochrane Risk of Bias Assessment tool was used for RCTs, and the Newcastle-Ottawa Scale (NOS) was used for observational studies.

Statistical Analysis

For the purpose of data analysis, we employed RevMan Version 5.4.1 (The Cochrane Collaboration, London, United Kingdom). We reported the risk ratio (RR) with a 95% confidence interval (CI) to compare the outcomes between the two study groups. A p-value of less than 0.05 was considered to indicate significance. Heterogeneity was assessed using I-square, and a threshold of I-square >50% was used to determine significant heterogeneity. In instances of notable heterogeneity, a random-effects model was applied to compare the outcomes. Otherwise, the analysis was conducted using a fixed-effect model. Subgroup analysis was performed based on study design (randomized and non-randomized studies).

Results

**Figure 1:**
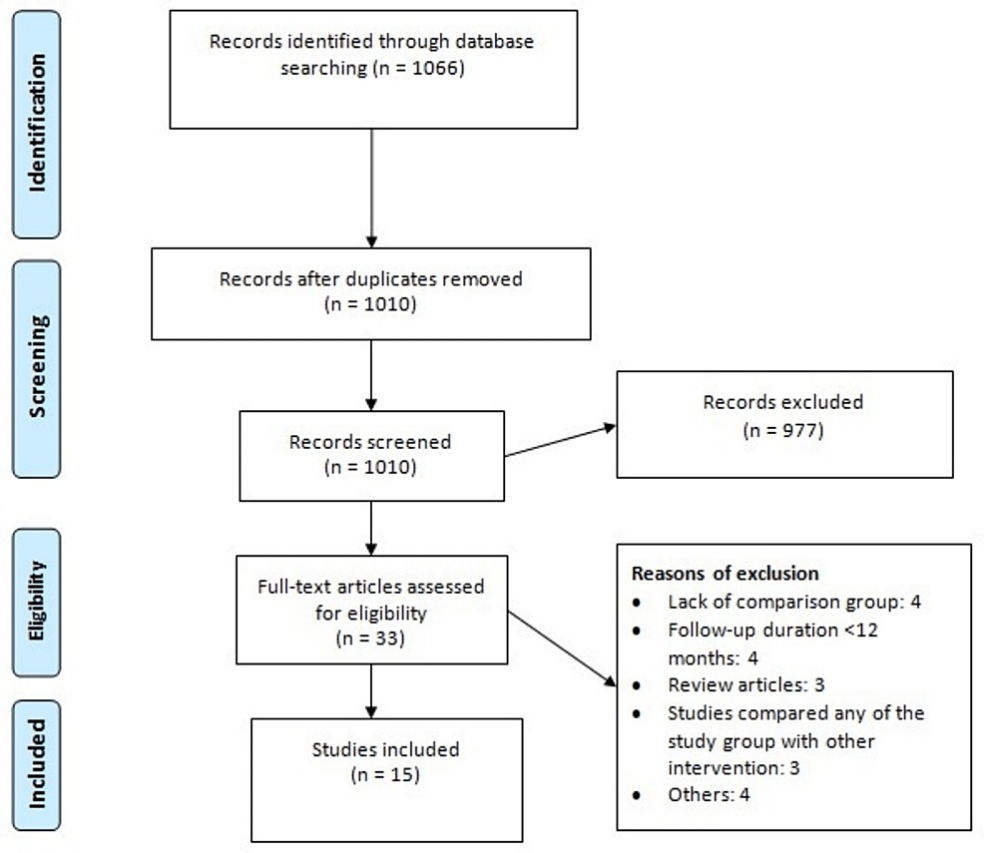
PRISMA flowchart of study selection

**Figure 2:**
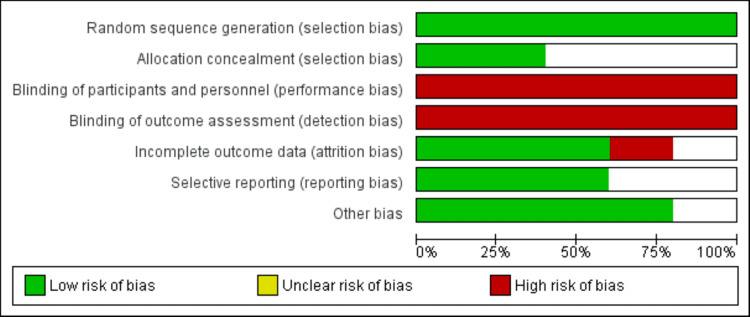
Risk of bias assessment of RCTs

Online database searches led to 1066 studies. As 56 studies were duplicates, we removed them before the initial screening. Initial screening was done for the remaining articles using their titles and abstracts. Out of 1010 studies, 33 studies were eligible for full-text screening. Finally, 15 studies fulfilled pre-defined eligibility criteria and were included in the final analysis. Figure 1 shows the PRISMA flowchart demonstrating the study selection process. Table 1 shows the characteristics of included studies. Out of 15 studies, 5 were RCTs. Follow-up duration of included studies ranged from 12 months to 54 months. Figure 2 shows the risk of biased assessment of RCTs. The quality assessment of observational studies is shown in Table 2.

**Table 1:** Characteristics of included studies. RCT: Randomized control trial; STEMI: ST-elevation myocardial infarction; NSTEMI: Non-ST segment elevation myocardial infarction (NSTEMI); DM: Diabetes Melitus; HTN: Hypertension; NR: Not reported

Author	Year	Study Design	ACS Type	Groups	Sample Size	Follow-up	Age (years)	Males n (%)	DM n (%)	HTN n (%)
Chung et al. [11]	2016	Observational	STEMI	Immediate	66	12 Months	63	48 (72)	38.3 (24)	34 (54.2)
				Staged	41		64	47 (74.1)	31.5 (20)	38 (59.3)
Diletti et al. [12]	2023	RCT	STEMI+NSTEMI	Immediate	764	12 Months	65.7	598 (78.3)	158 (20.7)	423 (55.4)
				Staged	761		65.3	589 (77.4)	163 (21.4)	395 (51.9)
Forero et al. [13]	2020	Observational	STEMI	Immediate	254	12 Months	66	181 (71.3)	12.6 (32)	101 (39.8)
				Staged	215		62	77.7 (167)	11.6 (25)	87 (40.5)
Iqbal et al. [14]	2017	Observational	STEMI	Immediate	1325	24 Months	65	1067 (80.5)	367 (27.7)	804 (60.7)
				Staged	658		64	509 (77.3)	137 (20.8)	342 (52)
Kakar et al. [15]	2023	Observational	STEMI+NSTEMI	Immediate	598	54 Months	66	450 (75.3)	100 (16.7)	259 (43.3)
				Staged	299		65	225 (75.3)	43 (14.4)	128 (42.8)
Kim et al. [16]	2014	Observational	STEMI	Immediate	67	36 Months	NR	NR	NR	NR
				Staged	252					
Kim et al. [17]	2017	Observational	STEMI	Immediate	316	40.8 Months	62.3	241 (76.3)	114 (36.1)	156 (49.4)
				Staged	360		63.2	263 (73.1)	117 (32.5)	189 (52.5)
Kornowski et al. [18]	2011	RCT	STEMI	Immediate	275	12 Months	62	218 (79.6)	42 (15.3)	151 (54.9)
				Staged	393		63.5	318 (80.9)	71 (18.1)	226 (57.5)
Maamoun et al. [19]	2012	RCT	STEMI	Immediate	42	12 Months	54.5	40 (95.2)	17 (40.5)	16 (38.1)
				Staged	36		52.3	32 (88.9)	20 (55.6)	12 (33.3)
Manari et al. [20]	2014	Observational	STEMI	Immediate	367	24 Months	66.7	258 (70.3)	71 (19.3)	219 (59.7)
				Staged	988		65.5	778 (78.7)	180 (17.5)	604 (61.1)
Mihnea-Traian et al. [21]	2021	Observational	STEMI	Immediate	50	12 Months	NR	NR	NR	NR
				Staged	50					
Mohamad et al. [22]	2011	Observational	STEMI	Immediate	7	12 Months	NR	NR	NR	NR
				Staged	12					
Park et al. [23]	2023	RCT	STEMI	Immediate	103	12 Months	63.3	82 (79.6)	42 (40.7)	56 (54.3)
				Staged	106		62.2	88 (83)	37 (34.9)	48 (45.2)
Sardella et al. [24]	2016	RCT	NSTEMI	Immediate	264	12 Months	72	207 (78.4)	98 (37.1)	193 (73.1)
				Staged	263		73	209 (79.5)	104 (39.5)	174 (66.2)
Yu et al. [25]	2016	Observational	NSTEMI	Immediate	291	36 Months	68	190 (65.3)	103 (35.4)	198 (68)
				Staged	291		69	192 (66)	94 (32.3)	199 (68.4)

**Table 2:** Quality assessment of observational studies.

Study ID	Selection	Exposure	Outcome
Chung et al. [11]	3	2	3
Forero et al. [13]	2	2	4
Iqbal et al. [14]	2	2	3
Kakar et al. [15]	3	1	2
Kim et al. [16]	3	2	3
Kim et al. [17]	3	2	4
Manari et al. [20]	3	2	3
Mihnea-Traian et al. [21]	2	2	3
Mohamad et al. [22]	3	1	2
Yu et al. [25]	3	2	2

Major Adverse Cardiovascular Events (MACE)

**Figure 3:**
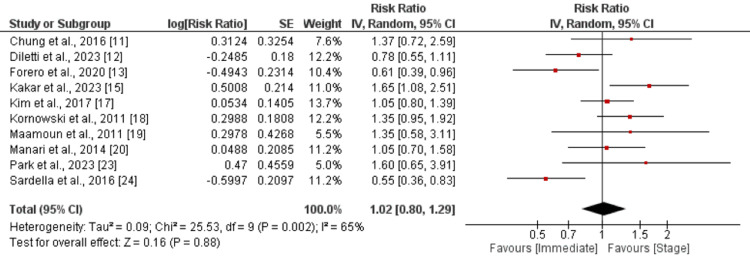
MACE Sources: References [11-13, 15, 17-20, 23-24]

The analysis of major adverse cardiovascular events (MACE) encompassed 10 studies. No significant difference was found between immediate and staged revascularization (RR: 1.02, 95% CI: 0.80-1.29), as shown in Figure 3. Significant heterogeneity was reported among the study results.

All-cause Mortality

**Figure 4:**
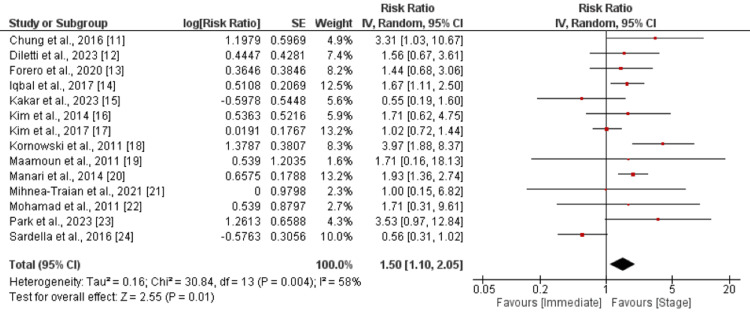
All-cause Mortality Sources: References [11-24]

The assessment of all-cause mortality involved a comprehensive review of 14 studies. As shown in Figure 4, the risk of all-cause mortality was 1.50 times higher in the immediate revascularization group compared to staged revascularization patients (RR: 1.50, 95%: 1.10 to 2.05). Significant heterogeneity was reported among the study results.

Cardiovascular Mortality

**Figure 5:**
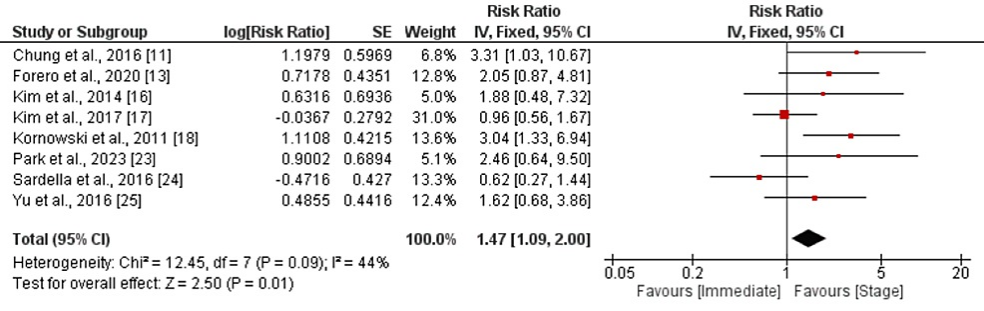
Cardiovascular Mortality Sources: References [11, 13, 16-18, 23-25]

Eight studies were included in the pooled analysis of the risk of cardiovascular mortality between patients in immediate and stage complete revascularization. As shown in Figure 5, the risk of cardiovascular mortality was significantly higher in an immediate group compared to its counterparts (RR: 1.47, 95% CI: 1.09 to 2.00). No significant heterogeneity was reported among the study results.

*Myocardial Infarction and *Revascularization

**Figure 6:**
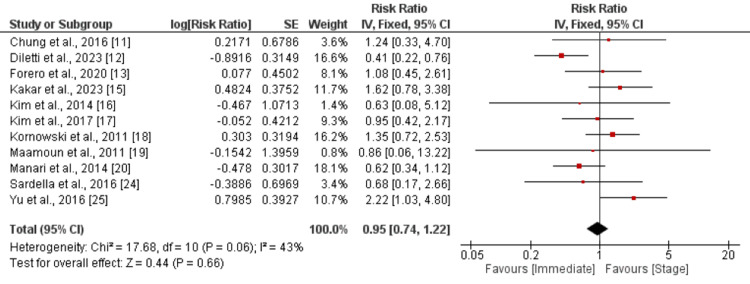
Myocardial Infarction Sources: References [11-13, 15-20, 24-25]

**Figure 7:**
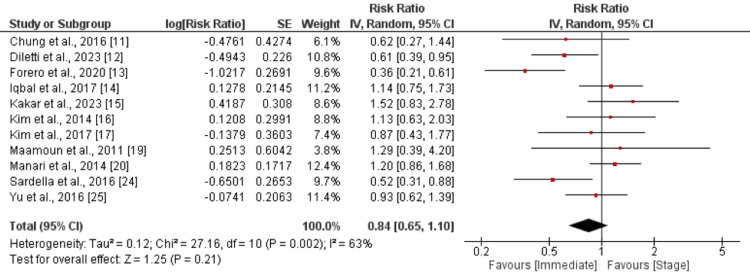
Revascularization Sources: References [11-17, 19-20, 24-25]

The pooled analysis focusing on myocardial infarction involved 11 studies. When comparing immediate revascularization with the staged approach, the calculated RR is 0.95, with a 95% CI ranging from 0.74 to 1.22, as shown in Figure 6. No significant difference was found between the two groups. Pooled analysis of 11 studies showed no significant difference between immediate and stage complete revascularization in terms of revascularization (RR: 0.84, 95% CI: 0.65 to 1.10), as shown in Figure 7.

Subgroup Analysis

The results reveal intriguing patterns in the analysis of outcomes across subgroups, as shown in Table 3. For major adverse cardiovascular events (MACE), within randomized controlled trials (RCTs), immediate revascularization demonstrates a lower risk in the immediate group (RR: 0.97, 95% CI: 0.64 to 1.46) despite a moderate degree of heterogeneity (I-square: 71%). In non-RCTs, the risk is slightly elevated in the immediate group (RR: 1.08, 95% CI: 0.79 to 4.46), but the differences overall non-significant All-cause mortality showcases a distinct contrast: RCTs exhibit significantly higher risk (RR: 1.52, 95% CI: 1.06 to 2.08) with minimal heterogeneity (I-square: 38%) in the immediate group, while non-RCTs also reported similar results (RR: 1.46, 95% CI: 1.10 to 1.92). Similar trends are seen in cardiovascular mortality and myocardial infarction, indicating potential differences in risk based on study type. Interestingly, revascularization rates within RCTs significantly favor immediate interventions (RR: 0.61, 95% CI: 0.44 to 0.84), with no observed heterogeneity (I-square: 0%), while non-RCTs depict a more modest difference (RR: 0.92, 95% CI: 0.68 to 1.25) alongside moderate heterogeneity (I-square: 64%).

**Table 3:** Subgroup Analysis MACE: Major adverse cardiovascular events; CV: Cardiovascular; RCT: Randomized control trial; MI: Myocardial infarction; CI: Confidence interval

Outcomes	Subgroup	RR (95% CI)	I-square
MACE	RCT	0.97 (0.64 to 1.46)	71%
	Non-RCT	1.08 (0.79 to 4.46)	62%
All-cause Mortality	RCT	1.52 (1.06 to 2.08)	38%
	Non-RCT	1.46 (1.10 to 1.92)	35%
CV Mortality	RCT	1.52 (0.89 to 2.61)	23%
	Non-RCT	1.45 (1.00 to 2.10)	18%
MI	RCT	0.74 (0.34 to 1.58)	58%
	Non-RCT	1.12 (0.76 to 1.66)	28%
Revascularization	RCT	0.61 (0.44 to 0.84)	0%
	Non-RCT	0.92 (0.68 to 1.25)	64%

Discussion

The current meta-analysis of 15 studies has found that in patients with ACS and multivessel CAD, staged revascularization is associated with improved outcomes, including all-cause mortality and cardiovascular mortality, without increasing the risk of major adverse cardiovascular events, myocardial infarction, and the need for unplanned revascularization.

Both meta-analyses and RCTs have consistently reported that complete revascularization is associated with improved outcomes in patients with ACS and multivessel CAD [26-28]. However, the optimal timing of complete revascularization remains unclear, with most of the data coming from observational studies only. A study conducted by Bainey and colleagues performed a meta-analysis showing that performing complete revascularization using MVI-S along with IRA PCI resulted in improved survival rates in both the short and long term while using MVI-I led to higher in-hospital mortality when compared to only IRA PCI [29]. A more recent network meta-analysis by Tarantini et al. found that MVI-S was linked to decreased short-term and long-term mortality compared to both IRA-only PCI and MVI-I. However, using only IRA PCI was associated with lower mortality rates than MVI-I [30]. Gaffar et al. conducted a meta-analysis exclusively focused on research contrasting prompt, complete revascularization with gradual complete revascularization within STEMI and NSTEMI patient groups.

Nevertheless, their selection criteria were limited to randomized controlled trials (RCTs), including only four RCTs encompassing a total of 853 patients. The immediate complete revascularization group exhibited notably reduced instances of unplanned repeat revascularization, alongside a suggestive inclination towards lower rates of major adverse cardiovascular events (MACE) [31]. In contrast to previous meta-analyses, we included NSTE-ACS as well as STEMI. Moreover, we included only those RCTs and observational studies in which staged and immediate complete revascularization were performed. This approach helped create a more homogeneous study population, allowing a true head-to-head pooled analysis. Additionally, our meta-analysis included recently conducted RCTs.

The present meta-analysis supports the use of staged revascularization in terms of safety. The reasons why multivessel intervention during the index primary PCI procedure may not be safe are unknown but are likely multifactorial. Any PCI procedure is challenging in the setting of hemodynamic instability and left ventricular dysfunction. The prothrombotic and inflammatory milieu in the early phase of STEMI may also increase procedural risks [32-33]. Second, lesion severity in non-culprit vessels can be overestimated during primary PCI because of diffuse coronary vasoconstriction and systemic endothelial dysfunction [34]. Third, multivessel PCI increases contrast use, which may be less tolerated in STEMI patients, especially if radiocontrast nephropathy develops [35]. Finally, unforeseen periprocedural complications in the non-culprit vessel may be poorly tolerated due to the "double jeopardy" of large myocardial territories at risk (i.e., simultaneous impairment of the culprit and non-culprit regions) [18].

The selection of an approach impacts treatment effectiveness and patient well-being and has implications for expenses and reimbursements. Implementing a staggered MV-PCI strategy leads to higher patient medical costs than an immediate MV-PCI approach [36-37]. Most national insurance committees, including those in China, tend to discourage using staggered PCI methods. Thus, cardiologists need to balance the economic downsides of staged MV-PCI against potential positive effects on patient outcomes. Surprisingly, in the current study, nearly half of the MV-PCI recipients opted for the staged approach, contrasting with the 21.2% rate reported in the Korean study [38]. There is an urgent requirement for compelling evidence to rationalize the additional expenses associated with staged MV-PCI. The present study highlights that a staged intervention could align with patients' best interests, providing a strong basis to advocate for staged interventions in cases of ACS and MVD. However, it is important to note that this study was observational and inherently limited; comprehensive, well-powered trials comparing these two strategies are essential.

Our meta-analysis supports the current recommendations for revascularization of the non-infarct-related artery in patients with ACS and multivessel disease. The optimal strategy for managing STEMI alongside multivessel disease involves pursuing comprehensive revascularization. To capitalize on survival advantages, begin with primary percutaneous coronary intervention (PPCI) targeting the infarct-related artery (IRA). Subsequently, adopt a consistent staged revascularization approach for stable non-IRA lesions to mitigate potential future adverse clinical occurrences. This staged PCI should ideally take place either during the initial hospital stay or within 45 days after the initial procedure, adhering to local protocols and available resources [39].

Our analysis is subject to several limitations. Firstly, variations in the timing of staged revascularization (MVI-S) across different studies introduced heterogeneity, preventing the identification of optimal timing. Secondly, we were unable to ascertain if specific patient subgroups experienced greater advantages from MVI-I compared to MVI-S. Thirdly, the absence of access to individual patient data hindered the possibility of conducting analyses based on patient-specific attributes. Lastly, discrepancies in the definition of multivessel coronary artery disease (CAD) and the interpretations of outcome measures like major adverse cardiovascular events (MACE) and repeat revascularization contributed to heterogeneity.

## Conclusions

In conclusion, this comprehensive meta-analysis provides valuable insights into the optimal timing for revascularization in patients with acute coronary syndrome (ACS) and multivessel coronary artery disease (CAD). The findings suggest that staged revascularization is associated with improved outcomes in all-cause mortality and cardiovascular mortality compared to immediate revascularization. No significant differences were observed in major adverse cardiovascular events or myocardial infarction between the two approaches. In clinical practice, these findings suggest that a staged revascularization approach, following an initial primary percutaneous coronary intervention targeting the infarct-related artery, could lead to better outcomes in patients with ACS and multivessel CAD. However, the decision-making process should consider individual patient factors, available resources, and local protocols.

## References

[1] Kolansky DM (2009). Acute coronary syndromes: morbidity, mortality, and pharmacoeconomic burden. Am J Manag Care.

[2] Baumann AAW, Mishra A, Worthley MI, Nelson AJ, Psaltis PJ (2020). Management of multivessel coronary artery disease in patients with non-ST-elevation myocardial infarction: a complex path to precision medicine. Ther Adv Chronic Dis.

[3] Dziewierz A, Siudak Z, Rakowski T, Zasada W, Dubiel JS, Dudek D (2010). Impact of multivessel coronary artery disease and noninfarct-related artery revascularization on outcome of patients with ST-elevation myocardial infarction transferred for primary percutaneous coronary intervention (from the EUROTRANSFER Registry). Am J Cardiol.

[4] Sorajja P, Gersh BJ, Cox DA (2007). Impact of multivessel disease on reperfusion success and clinical outcomes in patients undergoing primary percutaneous coronary intervention for acute myocardial infarction. Eur Heart J.

[5] Engstrøm T, Kelbæk H, Helqvist S (2015). Complete revascularisation versus treatment of the culprit lesion only in patients with ST-segment elevation myocardial infarction and multivessel disease (DANAMI- 3—PRIMULTI): an open-label, randomised controlled trial. Lancet.

[6] Smits PC, Abdel-Wahab M, Neumann FJ (2017). Fractional flow reserve—guided multivessel angioplasty in myocardialinfarction. N Engl J Med.

[7] Mehta SR, Wood DA, Storey RF (2019). Complete revascularization with multivessel PCI for myocardial infarction. N Engl J Med.

[8] Ibanez B, James S, Agewall S (2018). 2017 ESC Guidelines for the management of acute myocardial infarction in patients presenting with ST-segment elevation: The Task Force for the management of acute myocardial infarction in patients presenting with ST-segment elevation of the European Society of Cardiology (ESC). Eur Heart J.

[9] Collet JP, Thiele H, Barbato E (2021). 2020 ESC guidelines for the management of acute coronary syndromes in patients presenting without persistent ST-segment elevation. Eur Heart J.

[10] Dangas GD, George JC, Weintraub W, Popma JJ (2010). Timing of staged percutaneous coronary intervention in multivessel coronary artery disease. JACC Cardiovasc Interv.

[11] Chung WY, Seo JB, Choi DH (2016). Immediate multivessel revascularization may increase cardiac death and myocardial infarction in patients with ST-elevation myocardial infarction and multivessel coronary artery disease: data analysis from real world practice. Korean J Intern Med.

[12] Diletti R, den Dekker WK, Bennett J (2023). Immediate versus staged complete revascularisation in patients presenting with acute coronary syndrome and multivessel coronary disease (BIOVASC): a prospective, open-label, non-inferiority, randomised trial. Lancet.

[13] Tovar Forero MN, Scarparo P, den Dekker W (2020). Revascularization strategies in patients presenting with ST-elevation myocardial infarction and multivessel coronary disease. Am J Cardiol.

[14] Iqbal MB, Nadra IJ, Ding L (2017). Culprit vessel versus multivessel versus in-hospital staged intervention for patients with ST-segment elevation myocardial infarction and multivessel disease: stratified analyses in high-risk patient groups and anatomic subsets of nonculprit disease. JACC Cardiovasc Interv.

[15] Kakar H, Elscot JJ, De Gier A (2023). Propensity matched comparison of clinical outcome after immediate versus staged complete revascularization in patients with acute coronary syndrome and multivessel disease. Am J Cardiol.

[16] Chin CW, Le TT, Gao F (2014). Assessment of arterial elastance and ventricular-arterial coupling in patients with systemic lupus erythematosus. Int J Cardiol.

[17] Kim I, Kim MC, Jeong HC (2017). Optimal timing of percutaneous coronary intervention for nonculprit vessel in patients with ST-segment elevation myocardial infarction and multivessel disease. Korean Circ J.

[18] Kornowski R, Mehran R, Dangas G (2011). Prognostic impact of staged versus "one-time" multivessel percutaneous intervention in acute myocardial infarction: analysis from the HORIZONS-AMI (harmonizing outcomes with revascularization and stents in acute myocardial infarction) trial. J Am Coll Cardiol.

[19] Maamoun W, Elkhaeat N, Elarasy R (2011). Safety and feasibility of complete simultaneous revascularization during primary PCI in patients with STEMI and multi-vessel disease. Egypt Heart J.

[20] Manari A, Varani E, Guastaroba P (2014). Long-term outcome in patients with ST segment elevation myocardial infarction and multivessel disease treated with culprit-only, immediate, or staged multivessel percutaneous revascularization strategies: Insights from the REAL registry. Catheter Cardiovasc Interv.

[21] Mihnea-Traian NB, Popa V, Polojintef-Corbu DC, Popescu M (2021). Immediate versus staged complete revascularization in multi-vessel coronary artery disease patients with ST-elevation myocardial infarction uncomplicated by cardiogenic shock. Int Cardiovasc Res J.

[22] Mohamad T, Bernal JM, Kondur A (2011). Coronary revascularization strategy for ST elevation myocardial infarction with multivessel disease: experience and results at 1-year follow-up. Am J Ther.

[23] Park S, Rha SW, Choi BG (2023). Immediate versus staged complete revascularization in patients with ST-segment elevation myocardial infarction and multivessel coronary artery disease: results from a prematurely discontinued randomized multicenter trial. Am Heart J.

[24] Sardella G, Lucisano L, Garbo R (2016). Single-staged compared with multi-staged PCI in multivessel NSTEMI patients: the SMILE trial. J Am Coll Cardiol.

[25] Yu XF, Li Y, Wang QC (2016). Staged versus "one-time" multivessel intervention in elderly patients with non-ST-elevation acute coronary syndrome. J Geriatr Cardiol.

[26] Wald DS, Morris JK, Wald NJ (2013). Randomized trial of preventive angioplasty in myocardial infarction. N Engl J Med.

[27] Elgendy IY, Mahmoud AN, Kumbhani DJ, Bhatt DL, Bavry AA (2017). Complete versus culprit-only revascularization in patients with multi-vessel disease undergoing primary percutaneous coronary intervention: a meta-analysis of randomized trials. JACC Cardiovasc Interv.

[28] Dahal K, Rijal J, Panta R, Lee J, Azrin M, Lootens R (2014). Multi-vessel versus culprit-vessel and staged percutaneous coronary intervention in STEMI patients with multivessel disease: a meta-analysis of randomized controlled trials. Cardiovasc Revasc Med.

[29] Bainey KR, Mehta SR, Lai T, Welsh RC (2014). Complete vs culprit-only revascularization for patients with multivessel disease undergoing primary percutaneous coronary intervention for ST-segment elevation myocardial infarction: a systematic review and meta-analysis. Am Heart J.

[30] Tarantini G, D'Amico G, Brener SJ (2016). Survival after varying revascularization strategies in patients with ST-segment elevation myocardial infarction and multivessel coronary artery disease: a pairwise and network meta-analysis. JACC Cardiovasc Interv.

[31] Gaffar R, Habib B, Filion KB, Reynier P, Eisenberg MJ (2017). Optimal timing of complete revascularizationin acutecoronary syndrome: a systematic review and meta-analysis. J Am Heart Assoc.

[32] Ohashi Y, Kawashima S, Mori T, Terashima M, Ichikawa S, Ejiri J, Awano K (2006). Soluble CD40 ligand and interleukin-6 in the coronary circulation after acute myocardial infarction. Int J Cardiol.

[33] Kereiakes DJ, Gurbel PA (2008). Peri-procedural platelet function and platelet inhibition in percutaneous coronary intervention. JACC Cardiovasc Interv.

[34] Hanratty CG, Koyama Y, Rasmussen HH, Nelson GIC, Hansen PS, Ward MR (2002). Exaggeration of nonculprit stenosis during acute myocardial infarction: implication for immediate multivessel revascularization. J Am Coll Cardiol.

[35] Assali AR, Brosh D, Ben-Dor I, Solodky A, Fuchs S, Teplitsky I, Kornowski R (2007). The impact of renal insufficiency on patients' outcomes in emergent angioplasty for acute myocardial infarction. Catheter Cardiovasc Interv.

[36] Hannan EL, Samadashvili Z, Walford G (2013). Staged versus one-time complete revascularization with percutaneous coronary intervention for multivessel coronary artery disease patients without ST-elevation myocardial infarction. Circ Cardiovasc Interv.

[37] Blankenship JC, Moussa ID, Chambers CC, Brilakis ES, Haldis TA, Morrison DA, Dehmer GJ (2012). Staging of multivessel percutaneous coronary interventions: an expert consensus statement from the society for cardiovascular angiography and interventions. Catheter Cardiovasc Interv.

[38] Kim MC, Hyun JY, Ahn Y (2020). Optimal revascularization strategy in non-ST-segment-elevation myocardial infarction with multivessel coronary artery disease: culprit-only versus one-stage versus multistage revascularization. J Am Heart Assoc.

[39] Towashiraporn K (2022). Current recommendations for revascularization of non-infarct-related artery in patients presenting with ST-segment elevation myocardial infarction and multivessel disease. Front Cardiovasc Med.

